# Soft Attention Based DenseNet Model for Parkinson’s Disease Classification Using SPECT Images

**DOI:** 10.3389/fnagi.2022.908143

**Published:** 2022-07-13

**Authors:** Mahima Thakur, Harisudha Kuresan, Samiappan Dhanalakshmi, Khin Wee Lai, Xiang Wu

**Affiliations:** ^1^Department of Electronics and Communication Engineering, SRM Institute of Science and Technology, Chennai, India; ^2^Department of Biomedical Engineering, Faculty of Engineering, Universiti Malaya, Kuala Lumpur, Malaysia; ^3^School of Medical Information Engineering, Xuzhou Medical University, Xuzhou, China

**Keywords:** neural networks, Parkinson’s disease (PD), DenseNet architecture, region of convergence (ROC), area under the curve

## Abstract

**Objective:**

Deep learning algorithms have long been involved in the diagnosis of severe neurological disorders that interfere with patients’ everyday tasks, such as Parkinson’s disease (PD). The most effective imaging modality for detecting the condition is DaTscan, a variety of single-photon emission computerized tomography (SPECT) imaging method. The goal is to create a convolutional neural network that can specifically identify the region of interest following feature extraction.

**Methods:**

The study comprised a total of 1,390 DaTscan imaging groups with PD and normal classes. The architecture of DenseNet-121 is leveraged with a soft-attention block added before the final classification layer. For visually analyzing the region of interest (ROI) from the images after classification, Soft Attention Maps and feature map representation are used.

**Outcomes:**

The model obtains an overall accuracy of 99.2% and AUC-ROC score 99%. A sensitivity of 99.2%, specificity of 99.4% and f1-score of 99.1% is achieved that surpasses all prior research findings. Soft-attention map and feature map representation aid in highlighting the ROI, with a specific attention on the putamen and caudate regions.

**Conclusion:**

With the deep learning framework adopted, DaTscan images reveal the putamen and caudate areas of the brain, which aid in the distinguishing of normal and PD cohorts with high accuracy and sensitivity.

## Introduction

Parkinson’s disease (PD) is recognized as a chronic neurodegenerative condition of the central nervous system that primarily affects older adults (Pahuja et al., 2019) by Pereira et al. Researchers recognize the lack of dopaminergic neurons as the major cause of PD (Prediger et al., 2014). In the etiology of PD, oxidative stress is becoming a key factor in dopaminergic neuron degeneration (Zhou et al., 2009; Blesa et al., 2015). Loss of dopaminergic neurons is observed in substantia nigra of the mid-brain and later in loss of dopamine transporters in the striatum (Porritt et al., 2005). The striatum is the most significant component of the brain’s basal ganglia region, which produces is responsible for releasing the dopamine neurons in the mid-brain. The disease’s progressive nature may be attributed to the gradual deterioration in the striatum with age (Shahed and Jankovic, 2007).

Early PD is defined as the time frame before the start of severe motor symptoms and before the beginning of significant neurological impairment; yet, there is a scarcity of evidence that indicates the true potential of early therapy in terms of clinical and financial results (Zhou et al., 2009). More clarification is needed to study the true effect of early intervention on these outcomes. Future research should examine the impact of new diagnostic tools like genetic biomarkers on a wide range of medical issues (Porritt et al., 2005; Zhou et al., 2009).

Non-motor symptoms of PD result include anosmia (which affects the olfactory system), fatigue, disturbed sleep cycle, and fluctuations in bodyweight, disorders involving in temperament and cognitive aptitude, coronary artery disorders, bladder and bowel incontinence and digestive tract disorders. While the motor symptoms include resting tremor, rigidity, impaired body balance, slowing and freezing down of body movements or bradykinesia. As a result of such motor symptoms, the affected individual suffers from micrographia, dystonia and overall struggle in daily life activities. One may be able to find significant traits that are not normally employed in the clinical diagnosis of PD using machine learning algorithms, and depend on these alternative measures to diagnose PD in preclinical stages or atypical forms.

A person’s gait and movement patterns are closely scrutinized during a medical examination. Parkinson’s disease is characterized by bradykinesia (slow, tiny movements). Rigidity, or the quality of being rigid. During a medical exam, we passively move the patient’s joints to discover this. The arms, legs, and neck are often rigid in those with Parkinson’s disease. Restless tremors. While individuals aren’t paying attention or are preoccupied, these tremors come out, therefore a good opportunity to notice this is when someone is walking. To diagnose this illness, there are no biomarkers (tests or assessments). We don’t need to do imaging or laboratory testing unless we feel that there is a different reason for the patient’s symptoms. Movement indicators are just a portion of the picture for Parkinson’s disease, since it affects every region of the body.

Changes in memory and cognition, difficulty sleeping, emotional symptoms including worry and sadness, or even hallucinations, are among the most often reported symptoms. Patients with Parkinson’s disease are more likely to have issues with their autonomic nervous system. Controls such as heart rate, digestion, breathing, pupillary response and urine and sexual desire are all under the control of this system, which is mostly unconsciously active (Marine et al., 2019).

Neuroimaging technique in the recent past, particularly SPECT (single-photon emission computed tomography), have presented promising potential because of their sensitivity and specificity in diagnosing early PD. SPECT is proved to be more accessible to clinicians, being less expensive (Lauretani et al., 2015; Jinjin et al., 2019). The SPECT method of imaging avails 123I-FP-CIT, i.e., 123I-Ioupane. This radioligand binds the dopamine transporters in the striatum and termed as SPECT DaTSCAN Dopamine transporter levels in the brain may be seen using the DaTSCAN procedure, a form of Single-Photon Emission Computed Tomography (SPECT) (Harisudha et al., 2021). Traditionally, a standardized analysis and detection of such subject images are carried out by specialized technicians and radiologists. Notably, smaller putamen and caudate regions (the dopamine transporters) are observed in the case of PD patients, mainly because of the steady deficiency of dopaminergic neurons (Shahed and Jankovic, 2007; Mohammed et al., 2020).

In the healthcare industry, machine learning techniques are becoming more prevalent. Machine learning allows an algorithms to learn and extract meaningful representations from data in a semi-automated way, as the term indicates. Machine learning models have been used to diagnose Parkinson’s disease using a variety of data modalities, such as handwriting trends, gait patterns, and neuroimaging methodologies (Dhanalakshmi and Venkatesh, 2016; Matesan et al., 2018; Oláh et al., 2021).

Patients with PD who are diagnosed and treated early have decreased chance of progression and perhaps cheaper long-term care expenditures. Computer-Aided Diagnosis models that sufficiently make use of Artificial Intelligence (AI) techniques, particularly Deep Learning (DL) methods in the recent past; have suitably specialized as a reliable diagnostic tool (Matesan et al., 2018; Oláh et al., 2021). With the advancement in central processing unit (CPU) and graphics processing unit (GPU), better availability of reliable databases with ease of access in online platforms, and rapid improvisation of learning algorithms (Rumman et al., 2019; Bevilacqua et al., 2020).

Parkinson’s disease is distinct from other disorders in various ways, including how well it responds to levodopa. PD may be differentiated using a variety of neuroimaging methods, according to current scientific research. An imaging study using positron emission tomography (PET) has revealed a possible mechanism for the lack of response to PD treatment, as the study was also used in the preservation of dopamine receptors in PSP (Brooks et al., 1992). MRI with a high field strength (1.5 T) and a heavy T2 weighting, [18F]-fluorodopa positron emission tomography, [11C] raclopride imaging of dopamine D2 receptors, and single photon emission computed tomography of striatal dopamine reuptake sites are all possible imaging investigations (Warren et al., 2007).

MRI is the best structural imaging method that does not use ionizing radiation when compared to nuclear imaging. In the early stages of Parkinson’s disease, the vast majority of routine MRI methods failed to detect disease-specific abnormalities. Brain parenchyma sonography, a commonly used diagnostic tool for Parkinson’s disease (Brooks et al., 1992), recently revealed abnormal hyper echogenicity in both PD and essential tremor (Warren et al., 2007). As a recent research found that 77% of levodopa patients first reacted favorably to the medicine, levodopa has become an essential treatment for Parkinson’s disease (PD) (Brooks et al., 1992). Levodopa has been argued by physicians to be detrimental to prognosis since it is not definite of Parkinson’s disease (Warren et al., 2007). To distinguish Parkinson’s disease from other Parkinsonian illnesses, Apo morphine injections have been tried subcutaneously, however, they are ineffective and contribute very little to the diagnosis of PD (Brooks et al., 1992).

Despite promising pre-clinical data, many previously proposed medicines have failed clinical trials, underscoring the need of a well-thought-out study plan. Recent advances in our knowledge of the pathogenic processes and anatomical bases of Parkinson’s disease (PD) symptoms have opened up new therapeutic options, and it now seems likely that approaches to treating the disease will change considerably in the years ahead. Crediting the recent success of deep learning in medical image classification, this study relies on a similar motive, to detect the disease as early as possible, making optimum use of the convolutional neural network (CNN), a DL based architectural topology (Tagare et al., 2017; Rui et al., 2020).

In the case of making precise judgments based on large datasets, deep neural networks are clearly an asset. The methodology implemented in this research are Deep learning algorithms in which DenseNet 121 performed well when compared to other techniques. All previous layers provide extra input to DenseNet layers, which in turn provide their own feature maps to all following layers. Instead of adding the activations generated by one layer to the activations generated by subsequent levels, the activations are simply concatenated together. As the layers build upon one another, they share a “collective wisdom.” In order to maintain some kind of global state, the original inputs and activations from prior levels are retained at each layer (or, to be more accurate, between blocks of layers). A smaller number of parameters for a given depth is the result of this approach, which facilitates the reuse of existing features. Since dense networks can handle smaller datasets, they’re especially well-suited to them. Because there are no duplicate feature maps to train when DenseNets are connected in this manner, they need less parameters than a similar classic CNN. Some ResNets versions have also shown that many layers contribute little and may be eliminated. As a result, ResNets have a large number of parameters since each layer has its own weights to learn. A tiny number of new feature maps is all that DenseNets layers do, since they are relatively narrow (e.g., 12 filters). There was also an issue with training in extremely deep networks, due to the flow of information and gradients stated above. Because the gradients from the loss function and the original input picture can be accessed directly by each layer in DenseNets, this problem is alleviated (Ahlrichs and Lawo, 2013; Minja et al., 2019; Latha et al., 2020).

## Related Works

Rumman et al. (2019) proposed an image processing and Artificial Neural Network (ANN) based approach to find the domain of putamen and caudate as the region of interest from SPECT images for detecting PD in its early stage. The region values of the putamen and caudate were then fetched to the ANN classifier for recognition.

Wolfswinkel et al. (2021) developed a convolutional neural network called DaTNet-3 to differentiate and classify normal and PD subjects that underwent the DaTSCAN procedure. They collected the imaging data from Parkinson’s Progression Marker Initiative (PPMI) and a hospital-based dataset. Wenzel et al. (2019) explored variable image characteristics at different camera settings using FP-CIT SPECT to train the InceptionV3 CNN model for automated classification. Three image settings: unsmoothed, smoothed, and combination of smooth and unsmoothed were fed into the neural network.

Dhanalakshmi et al. (2019) introduced the role of isosurface to extract and collect only the most relevant features from complex 3D DaTSCAN images. This method was further utilized to implement CNN architectures such as LeNet and ALexNet for PD classification. Martínez-Murcia et al. (2017) performed an exhaustive analysis of DaTSCAN images implementing a voxel-based logistic lasso model. The model helped to define the regional voxels in the caudate, putamen, and globus pallidus area for an informed classification of control and PD categories. Additionally, another ML technique called logistic component analysis was utilized for judging feature differences within the same population or groups.

Ortiz et al. (2019) utilized Alexnet architecture and introduced an image normalization layer to capture the region of interest from SPECT images. The model helps achieve high classification accuracy for classifying PD and control groups. Adams et al. (Subhrajit et al., 2018) performed a quantitative analysis of DAT SPECT imaging by combining the baseline score of DAT image scans with UDPRS_III (motor function scores) as base input parameters. These features, which included motor function and DAT scan scores, were then provided as input to the CNN model for prediction and classification for PD.

Oliveira et al. (Mohammed et al., 2020) assessed certain features that contribute to dopaminergic degeneration for PD using [123l] FP-CIT SPECT brain scans. A total of seven features were calculated and employed for the assessment using ML classifiers that include Support Vector Machine (SVM), K-Nearest Neighbours (KNN), and Logistic Regression (LR). Most accurate results were obtained using the SVM classifier with the seven features.

Martínez-Murcia et al. (Oliveira et al., 2018) developed and proposed a 3-D CNN system for a fast feature diagnosis of PD using SPECT imaging modality. Activation maps were constructed and visualized for practical feature understanding of the network. Shiiba et al. (2020) studied and assessed shape features of SPECT images combined with semi-quantitative parameters to feed into Machine Learning classifiers. The semi-quantitative features and the shape features were used to extract and study the region of interest in classifying PD.

Pianpanit et al. (2021) investigated different model interpretation methods using SPECT images with deep learning model approaches. Techniques like SHAP (Shapley Additive explanations) and guided backpropagation were explored for their attributes and their performance compared for distinguishing between normal and PD subjects.

It is critical to diagnose PD at an early stage, given that the severity of PD and its many phases are essential in determining when to intervene. Many researchers have suggested a model predict and diagnose the disease. Deep learning for image analysis has yielded some of the most impressive progress in recent years (Adams et al., 2018). Various deep learning and machine learning approaches have been used to predict PD in multiple studies. Imaging modalities including MRI (Magnetic Resonance Imaging) and SPECT for detecting PD have raised high interest in research studies.

Sivaranjini et al. (Adams et al., 2018) attempted to classify PD and healthy control images from MRI modality by following the architecture of the transfer learning model, AlexNet. This model successfully recognizes the structural differences in normal and PD subjects and yields optimal results. Chakraborty et al. (Magesh et al., 2020) carried out data pre-processing for 3T T1-Weighted MRI brain scans and designed a 3D CNN model for extracting complex patterns in the brain images of normal and PD cohorts.

All studies that employed accuracy in model assessment obtained a diagnostic accuracy above chance values for each research. It’s possible certain data types may not be generalizable enough to forecast how effectively they may help us discriminate between Parkinson’s disease and other Parkinsonian illnesses, but the application of machine learning to many various kinds of data led to great diagnostic accuracy in PD. Data splitting procedures and cross validation were not described despite the great diagnostic accuracy and performance reported in many investigations. When 2D slices are derived from 3D volumes in data modalities like 3D MRI scans, several slices may be created for a single patient. Data leakage and an overestimation of model performance may occur if the same subject is used in the training, validation, and testing sets. This compromises the repeatability of reported findings.

In the proposed work the following approaches are taken as objectives for this study:

1. Taking the PPMI repository’s DaTSCAN SPECT images as the adequate dataset for usage in the study.
2. Comparing healthy Vs. PD cohorts contour edge imaging technique.
3. Analyze and train the images on DenseNet-121, CNN topologies with a soft attention block in addition to it, influenced by the works of Martínez-Murcia et al. (2017), Oliveira et al. (2018), and Wolfswinkel et al. (2021).
4. Compare the results with other pre-trained CNN models.
5. Visualize the successful model results using Soft Attention Map and Feature Map representation.
6. Analyze the results using statistical metrics.

The paper is structured as follows: The section 3 explains about the materials and methods followed by section 4 deals with the methodology in detail along with the results and discussion. The session 5 concludes the research work.

## Materials and Methods

### Dataset

The data for this research was acquired from the PPMI public repository, which is a multimodal, prolonged study of radiomic feature observations, neuroimaging, and biological markers in PD patients and healthy controls (HC).

Various industries’ scientists, researchers, sponsors, and study populations have continued to work together to build this substantial searchable archive to make PD research and therapies easier and more effective by finding progression biomarkers.

### Data Preprocessing

A SPECT scan generates a volumetric image of the basal ganglia. Typically, a collection of axial view planes is then created for clinical evaluation. These picture sequences have been anonymized and exported in PNG format. All images were created using a single slice that is most typical of the basal ganglia’s anatomical location.

**FIGURE 1:**
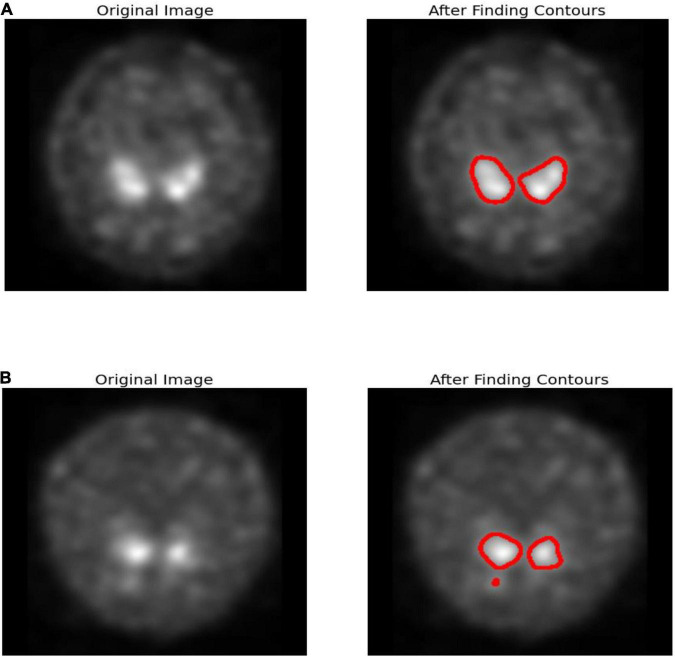
Edge detection **(A)** healthy subject **(B)** PD subject.

To begin, the image is first pre-processed and the ROI, the putamen and caudate area, is segmented. The caudate and putamen area segmentation areas are computed and given as features to the Deep learning algorithms. With the help of the training data, the DL algorithms are trained and the prediction model may be utilized to distinguish PD patients from healthy ones. The deep learning algorithms implemented in this research work are DenseNet 121, Xception, ResNet 50, MobileNet V2, Inception ResNet V2, ResNet 152V2, EfficientNet B1. The Figure 1 depicts the suggested procedure using SPECT pictures from the PPMI database.

#### Contour Edge Detection

Edge detection is a technique for detecting the borders of objects in images. It detects brightness disparities in image processing, computer vision, and machine vision fields. Edge detection is used to extract images and data. Edge detection are essential in computer vision since they involve identifying and classifying objects in images.

In Parkinson’s disease, nigrostriatal loss is often disproportionate, with greater degradation observed in the putamen relative to the caudate nucleus. Corresponding with Parkinson’s disease are aberrant appearances such as symmetrical loss of uptake in both putamen and total loss in absorption in the caudate and putamen despite usual functioning. Figure 1 uses canny-edge detection to highlight the putamen and caudate region in normal and healthy cohorts. Finding a closed form and drawing the object’s border is the primary goal of contour detection as shown in Figure 1. It is also possible to employ contour detection to estimate an object’s form based on such attributes as its aspect ratio, length, and solidity. The images are accustomed to working with grayscale images, the first step is to transform the image to gray. To approximate contours, a simple threshold is utilized. Smoother contours may be achieved by using the OPEN and CLOSE technique. A list of contours is obtained and the final contours are sketched on the color.

#### Data Augmentation

**FIGURE 2:**
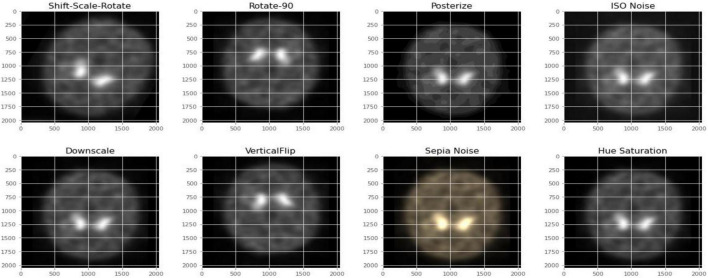
Data augmentation for healthy control participants.

**FIGURE 3:**
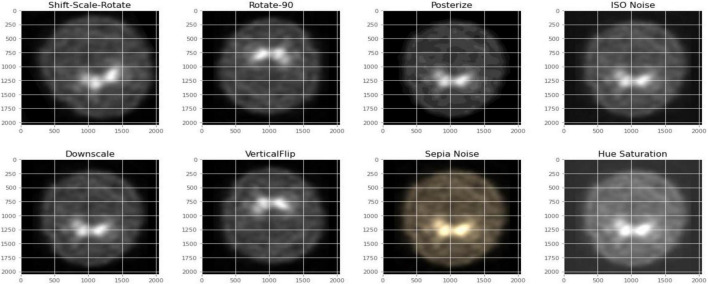
Data augmentation for PD participants.

Figures 2, 3 depict the data augmentation that was performed on both the subtypes, Health Control and PD patients. Data augmentation was adopted to correct the balance of the dataset due to its moderate size and the small number of HC participants engaged in the screening procedure. The description of the eight different type of augmentations is mentioned in the Table 1. The eight augmentation techniques were so chosen manually that the model extracts different spatial representations throughout the dataset in order to ensure that the final models function effectively in the event of several limitations like over fitting conditions. After data augmentation, 1,840 images of HC participants and 2,002 images of PD patients are generated. The imaging data selected for this study includes 1,390 DaTscan SPECT images, which are split into two classes: PD (with 1,160 images) and Healthy Controls (with 230 images), as shown in Table 2. The PPMI imaging support validated the diagnosis of PD by confirming that the screening DaT-SPECT (123I FP-CIT) is associated with a DaT deficiency. The network is divided into two segments during the training phase. The augmentation network uses two images of the same kind as the input image and outputs a layer with the same resolution as the input image. This layer is used to create an “enhanced” version of the original picture. Finally, the enhanced picture is sent into a second network, which is called a classification network. An end-of-network drop in classification accuracy is due to a loss in cross entropy on class sigmoid. End-to-end, an addition loss is calculated to control how well augmented images match their input counterparts. As a result, the total loss is the sum of these two losses.

**TABLE 1:** Image augmentation types and its description.

Augmentation type	Description
Shift Scale Rotate	Shifts along the x/y axis, scale (zoom in/out) and rotates on a random value
Rotate-90	Rotate by 90 degrees.
Posterize	Reduces the number of bits for each color channel
ISO Noise	Random sensor noise or Gaussian noise.
Downscale	Reduces the overall resolution of the image
Vertical Flip	Flip the input vertically around the x-axis.
Sepia Noise	Sepia filter is added randomly
Hue Saturation	Adds hue saturation to the image

**TABLE 2:** Dataset of DaTscan SPECT images.

Study class	No. of subjects	Average age	
		Male	Female
PD	1160	63.68 ±	62.35 ±
CN	230	61.87 ±	59.26 ±

**FIGURE 4:**
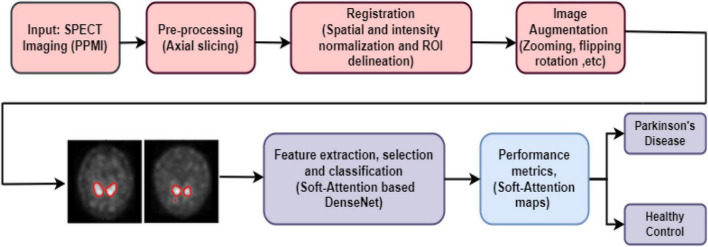
Overall block diagram of the proposed work.

The overall block diagram of diagnosis of PD using SPECT images is shown in Figure 4. The SPECT image is pre-processed, augmented and classified using various Deep learning algorithms. The performance metrics are compared for with and without augmented images. The metrics are accuracy, sensitivity, specificity, precision and F1 score. The SPECT quantitation of a given picture feature is affected by a wide range of physical parameters, but three stand out: attenuation, scatter and detector response (or finite spatial resolution limited by the collimator). As the image feature size falls, detector response, or limited spatial resolution, becomes more critical in SPECT quantification.

The detected activity concentration drops with the volume of features smaller than nearly twice the detector’s spatial resolution. This is because the SPECT image’s count values are dispersed across a broader area than the emission source itself. As a result, the actual concentration is lowered. If a source is big enough, the dispersion of counts away from that source is counterbalanced to a greater extent than with smaller sources. Linear deconvolution filtering, such as Wiener or Metz filters, may be used to adjust detector response. The detector response will become blurry if the filter gain is greater than unity at low spatial frequencies. High-frequency picture noise may be controlled by “rolling-off” the filter to zero gain. The need for a model to display what location it is attending to while making a decision/prediction in deep learning. Various attention mechanisms have been introduced in the past years. With the development of automated pattern learning mechanisms, particularly models that can be trained to focus on specific regions, it is now possible to focus on critical areas for attention.

## Methodology

### DenseNet Architecture

The DenseNet (densely connected convolutional network) is recognized for having convolutional neural network architecture that is state-of-art, when validated for classification using the popular ImageNet dataset. Huang et al. validated the technique of using direct connections in a feed-forward manner from each layer to every other layer. Every layer in the model architecture takes the target input and concatenation of the preceding layers’ feature maps. It performs non-linear operations such as batch normalization, ReLU, and convolution or pooling. The resultant feature maps of each layer are provided as inputs to the succeeding connected layers after the non-linear function’s computation. If the size of the feature maps changes, the concatenation procedure is unsuccessful. Hence, the need for pooling operation is crucial when the size of the feature maps varies (Chakraborty et al., 2020).

The architecture is organized into distinct blocks, i.e., Dense blocks (densely connected) to assist in the pooling process. The layers between dense blocks are transition layers that conduct the tasks of convolution, batch normalization, and pooling. On an average note, each function generates K unique feature maps, a hyper-parameter known as the growth rate which determines the number of feature maps each layer delivers to the network (Sivaranjini and Sujatha, 2020). Once updated, the feature maps may be viewed throughout the network. Unlike other traditional CNN models, this also waives the need to reproduce one layer to another.

**FIGURE 5:**
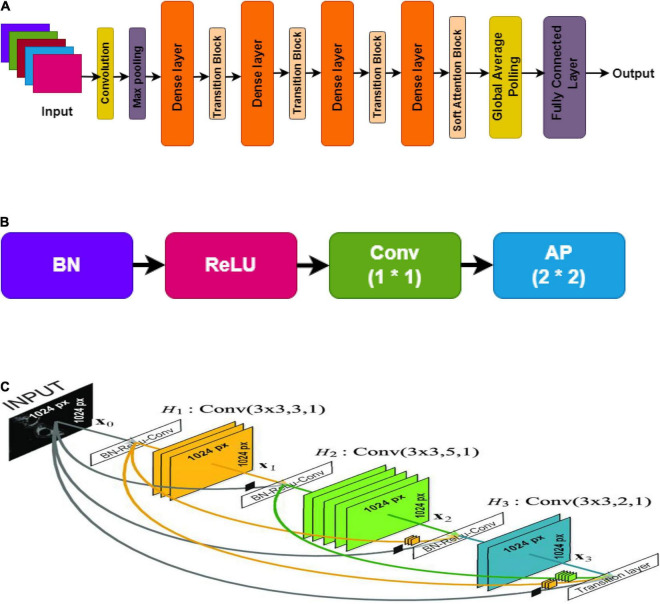
DenseNet, **(A)** overall architecture; **(B)** transition layer; **(C)** dense layer.

Each layer in the network reproduces k feature maps and causes many parameter inputs as shown in Figure 5. As a solution, to limit the number of input feature maps to 4k, a [1 × 1] size of convolution was employed in the bottleneck layer. Thus, minimizing the amount of feature mappings at transition layers is another optimal feature of DenseNet. The number of feature maps in a dense block with n feature maps results in θn, later in the transition layer, lying in the factor range of 0 < θ ≤ 1, known as the compression factor (Gao et al., 2019). DenseNet’s design provides many advantages in addition to network compactness: it overcomes the vanishing gradient problem, optimizes feature transfer, and minimizes the rate of parameters. DenseNet121 network architecture was utilized in this paper.

### Soft Attention Block

SPECT DaTSCAN method helps in distinguishing between Parkinson’s and control subjects by helping to visualize the basal ganglia region. The dopamine transporters: putamen and caudate regions are reported to get smaller in size due to the loss of dopaminergic neurons in the case of PD patients. Soft attention can be a useful idea to detect the region in the image where minor to significant distortion is found, which is considered an abnormality and needs further analysis.

**FIGURE 6:**
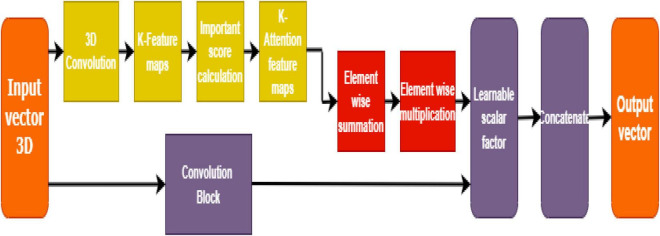
Soft attention mechanism.

Soft attention takes the robust approach of promoting the most relevant input (in this case, pixels in an image) while still allowing a subset of the other information to contribute to the model’s decision-making as shown in Figure 6. It is taking inspiration from the good works of Martínez-Murcia et al. (2017) and Dhanalakshmi et al. (2019), a soft attention block that utilizes 3D-convolution to attend and identify the essential features responsible for classification.

This way, the high-level features are first extracted, and the resultant convolution of ‘K’ kernels generates a feature map (having K attention heads). This feature map is further normalized to calculate the importance score (or *soft attention* score) based on the appropriate location from the extracted feature map. A 3-D tensor *f^x^* ∈ ℝ*^hx^*×*^w^^x^*×*^d^^x^* having a 3-D kernel of size *H* ∈  3*x* 3*xn^x^* is input to the convolutional layer that results in a feature map*f*_3*d*_ ∈ ℝ^*h*^*^x^*×^*w*^^*x*×1^.

Such K kernels, work as attention points to generate *f*_3*d*_ ∈ ℝ^*h*^*^x^*×*^w^^x^*×*^K^*. These feature maps are further normalized and averaged to be retained for calculating the importance score (for dominant locations) or the soft attention score. The equation for soft attention score denoted by “*S*” is given below:


(1) $$S=\sum_{k=1}^{K}\frac{\exp\left(f_{3\,d_{i\,j}}\right)}{\sum_{i=1}^{w^{x}}\sum_{j=1}^{h^{x}}\text{exp}\,\left(f_{3\,d_{i\,j}}\right)};\mathrm{where}\,f_{3\,d}=H\,\left(f^{x}\right)$$

The resultant *f^x^* tensor is thereby multiplied with the soft attention score S, so the value becomes$f_{s}^{x}$. A learnable scalar is assigned to compute the weights, which in this case is *y* with a value of 0.01. The total weight is calculated by the equation given below:


(2) $$\alpha_{y}=f^{x}+y\,f_{s}^{x}$$

The finalized soft attention layer helps the model decide the specific locations of the feature map that has important attributes on the whole.

Figure 5 shows the soft attention maps.

Convolution and pooling are the foundation of DenseNet. In order to get to the classification layer, there are four more dense blocks followed by transition layers. After that comes a dense block followed by yet another transition layer. The DenseNet121 architecture with Soft Attention Block for classification of PD and HC cases is shown in Table 3.

**TABLE 3:** DenseNet121 architecture with Soft Attention Block for classification of PD and HC cases.

Layers	Output shape	Kernel size and details
Convolution 2D	112×112	7× 7*conv*, stride 2 (Rui et al., 2019)
Max Pooling 2D	56×56	3×3 *max*−*pool*,*stride* 2
Dense Block (Pahuja et al., 2019)	56×56	$\left[\begin{array}{c} 1\times 1\text{conv}\\ 3\times 3\text{conv} \end{array}\right]\times 6$
Transition Layer (Pahuja et al., 2019)	56×56	1× 1*conv*
	28×28	2×2*average pool*, *stride* 2
Dense Block (Prediger et al., 2014)	28×28	$\left[\begin{array}{c} 1\times 1\text{conv}\\ 3\times 3\text{conv} \end{array}\right]\times 12$
Transition Layer (Prediger et al., 2014)	28×28	1× 1*conv*
	14×14	2×2*average pool*, *stride* 2
Dense Block (Blesa et al., 2015)	14×14	$\left[\begin{array}{c} 1\times 1\text{conv}\\ 3\times 3\text{conv} \end{array}\right]\times 24$
Transition Layer (Blesa et al., 2015)	14×14	1× 1*conv*
	7×7	2×2*average pool*,*stride* 2
Dense Block (Zhou et al., 2009)	7×7	$\left[\begin{array}{c} 1\times 1\text{conv}\\ 3\times 3\text{conv} \end{array}\right]\times 16$
Soft Attention Block	7×7	*SoftAttention*× 1
Classification	1×1	7× 7*global average pool*
Layer	2	*Fully Connected Dense Layer*, *Softmax*

The stride is 2 and the first convolution block comprises 64 filters of size 7 × 7. After that, there’s a MaxPool layer with 3 × 3 max pooling and a stride of 2. ReLU activation and the real Conv2D layer follow BatchNormalization in every convolutional block. In Table 3, convolutions with 1 × 1 and 3 × 3 kernel sizes are used in each dense block. This is repeated six times in dense block 1, twelve times in dense block 2, twenty-four times in dense block 3, and ultimately sixteen times in dense block 4. Each 1 × 1 convolution has four times the number of filters in dense block. As a result, 4 filters are employed, yet only 3 of those filters are ever used. In addition, the input tensor and the output tensor must be joined.

The number of channels in the transition layer is to be reduced to half of the current channels. An average pool layer with a stride of two is used in conjunction with a 1 × 1 convolutional layer. bn rl conv already has a kernel size of 1 × 1, therefore we don’t need to declare it again.

Half of the channels in the transition layers must be removed. To figure out how many channels there are, we need to acquire half of the input tensor x. As a result, we may utilize Keras backend (K) to produce a tuple with the dimension of x when given a tensor x. For our purposes, we simply need to know how many filters there are in that form. So [−1] is added. This number of filters may be divided by two to reach the desired result.

The dense blocks and transition layers have now been defined. The thick blocks and transition layers must now be stacked one on top of the other. Since the repetitions are 6,12,24,16 we build a “for loop” to go through them. In this way, the loop is executed four times, each time with a different number from the range of 6, 12, 24, or 16. The dense blocks and transition layers are now complete.

There is a final output layer, then Global Average Pooling. Following Dense Block 4, there is no transition layer between Dense Blocks 3 and 4, but it goes straight into the Classification Layer after Dense Block 4. Global Average Pooling is used on the connection ‘d,’ not the one on ‘x,’ as was previously stated. To eliminate the for loop from the above code and stack the levels one after the other without a transition layer is another option.

## Results and Discussion

### Visual Assessment

**FIGURE 7:**
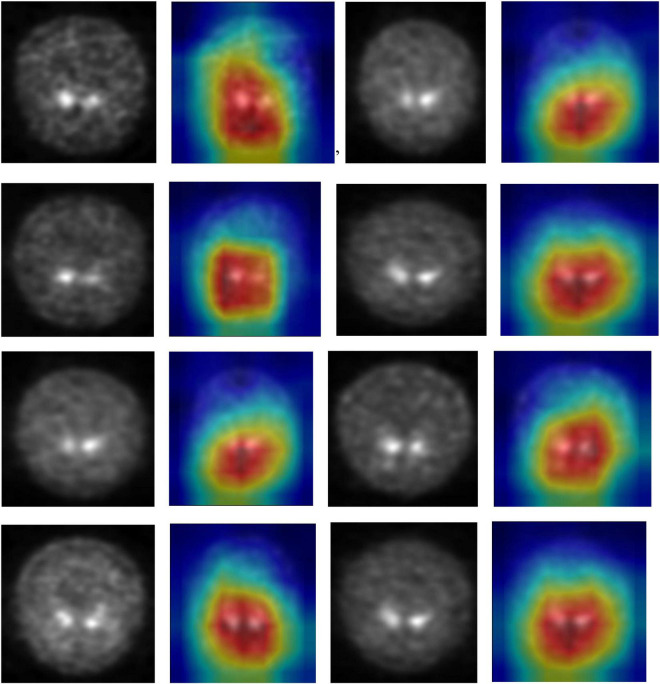
Soft attention map visualisation of PD patients with the ROI highlighted.

**FIGURE 8:**
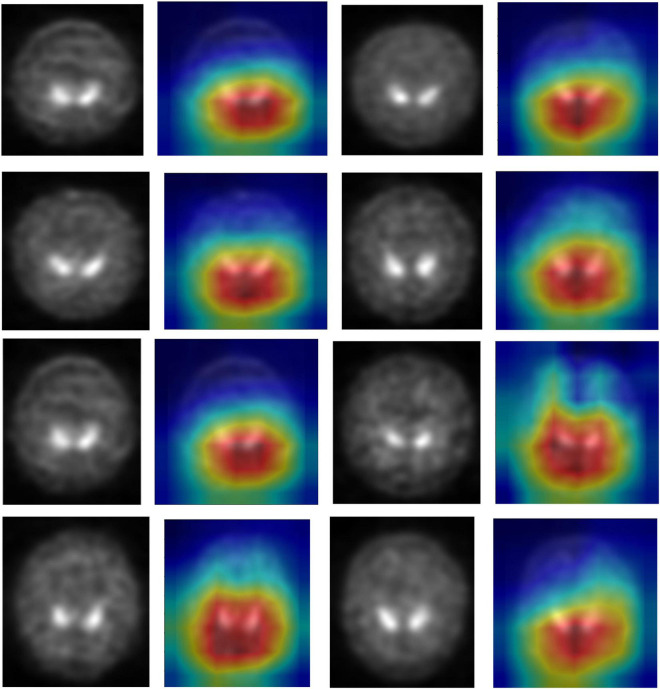
Soft attention map visualisation of HC patients with the ROI highlighted.

DenseNet is compared with the other deep earning algorithms such as ResNet, Inception ResNet, Xception, MobileNet, and EfficientNet V2. The vanishing gradient issue was solved by introducing the idea of residual connections in ResNet V2. Inception ResNet, a ResNet version that employs several size kernels inside the same layer, is utilized since it is difficult to select on a ResNet kernel size. For example, Xception proposed the notion of depth-wise separable convolution in order to minimize the number of parameters without compromising performance. There are now between 100 and 1,000 less parameters since MobileNet has included point wise convolution in addition to depth wise convolution. The Soft Map visualization for PD and healthy controls are shown in Figures 7, 8. In soft attention, instead of utilizing the image x as an input, we use weighted image characteristics compensated for attention in soft attention. The areas of the image that get the most attention seem brighter. The weighted characteristics of the DL algorithms, as well as the PD and normal it predicted, are shown in the image above. The low weight of the feature map multiplied by the soft focus discredits places that aren’t significant. As a result, regions with high levels of attention retain their original worth while those with low levels of attention approach zero (become dark in the visualization). With the “PD and normal patient,” the attention module creates a new feature map with all areas darkened except the region of interest area.

In order to know the working of the model, and how it defines the ROI through its various layers, feature maps visualization may be useful. Figure 8 shows layers inside the dense block, including batch normalization and ReLU activation, and how they influence in the overall classification work of the model. These feature maps highlighted help in conclusively deciding the putamen and caudate regions taking the major role in predicting the desired class.

**FIGURE 9:**
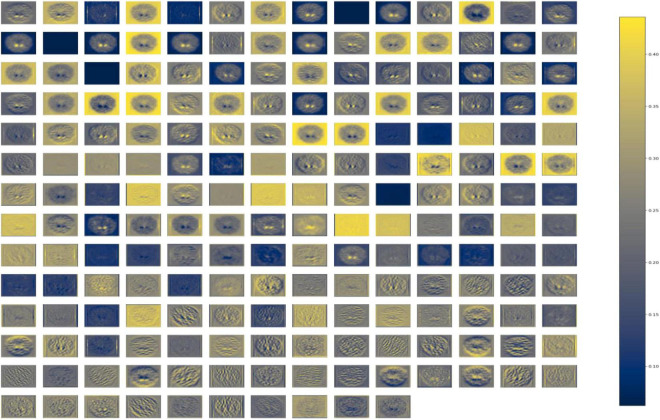
Dense Block of DenseNet121 with batch normalization operation.

The Figure 9 represents the DenseNet 121 algorithm implementing Batch normalization operation. Normalizing network activations over a mini-batch of a certain size is what batch normalization is all about. It is possible to normalize a mini-batch of data by computing the mean and variance for each characteristic. To get the feature’s standard deviation, remove the mean and divide the feature by the mini-batch standard deviation. Batch normalization enhances the model’s training speed by smoothing the loss function and improving the model’s parameters. Poorly initialized neural networks are addressed by batch normalization. Pre-processing may be done at every level of the network, according to this interpretation. At the start of training, it compels the activations in a network to take on a unit Gaussian distribution. One of the most often used techniques for training deep neural networks (DNNs) is Batch Normalization (BatchNorm). The gradients are more predictable and steady as a result of this smoothness, making it easier to train.

Internal covariate shift is no longer an issue. This ensures that each layer’s input is spread around a common mean and standard deviation. Assume for a moment that we’re training an image classification model that sorts photos into one of two groups: PD or NPD. If we just have photographs of PD, these images will also be distributed in a certain manner. The model’s settings will be updated as a result of using these photographs. if we get a fresh batch of photos from people who are not diagnosed with PD. As a result of this change, the distribution of these new photos will be somewhat altered. Using these fresh photos, the model will adjust its parameters. As a result, the distribution of the concealed activation will shift as well. It’s called an internal covariate shift, and it’s a change in the concealed activation. Batch normalization enhances the model’s training speed by smoothing the loss function and improving the model’s parameters.

**FIGURE 10:**
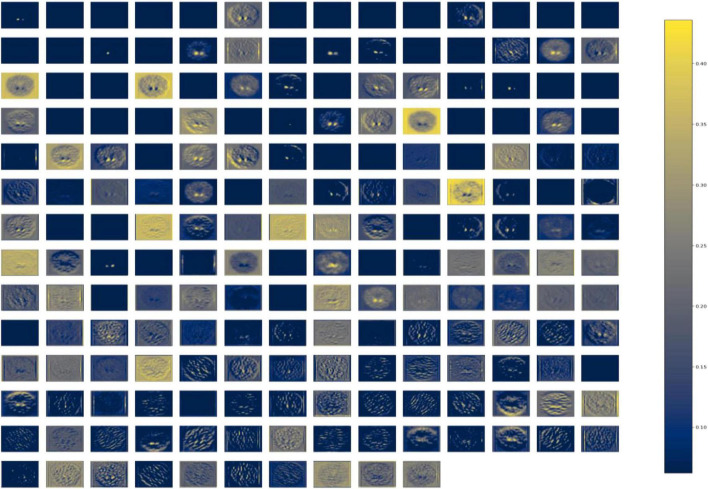
Dense Block of DenseNet121 with ReLU activation.

Figure 10 represents the DenseNet 121 algorithm implementing ReLU activation function. It is possible to increase the learning pace of deep neural networks by using ReLU activation functions in the hidden layers. Deep neural networks now employ the rectified linear unit as their typical activation function. Using ReLU activation function, the vanishing gradient issue is avoided. This is the reason why the deep neural network’s learning speed can be improved by activating ReLU. As a result of avoiding the need to do exponential and division computations, employing rectified linear units speeds up computations significantly. Squeezing values from 0 to the maximum imparts sparsity into the hidden units, another ReLU feature. ReLUs may readily overfit when compared to sigmoid functions, although the dropout approach has been used to mitigate this problem, and deep neural networks with corrected networks have shown enhanced performance. Because of its simplicity and dependability, the ReLU and its derivatives have been included into several deep learning systems.

### Quantitative Assessment

**FIGURE 11:**
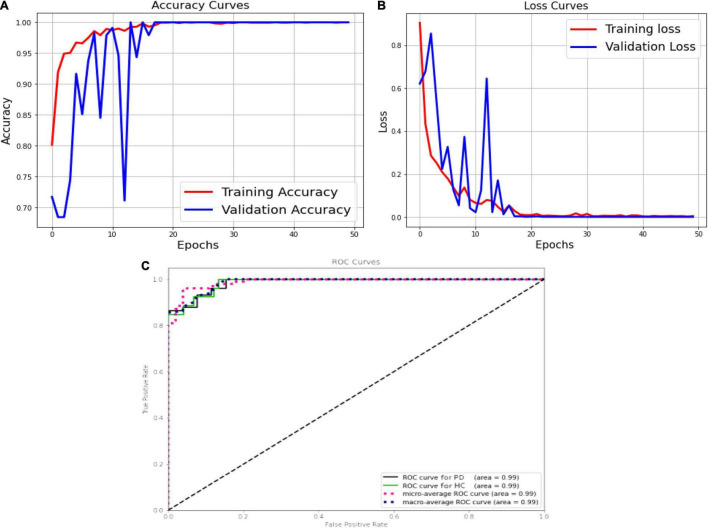
DenseNet, **(A)** accuracy; **(B)** loss curve; **(C)** ROC curve.

The Figure 11A shows the validation accuracy plot having an accuracy of 99.2% and the validation loss is shown in Figure 11B. An AUC of 99% is achieved for DenseNet 121 architecture. Classification methods rely on the AUC-ROC statistic to gauge their effectiveness is shown in Figure 11C.

**FIGURE 12:**
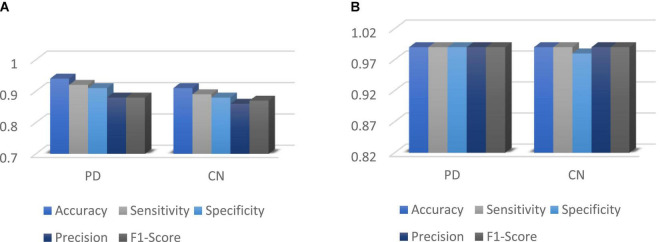
**(A)** Without augmented images; **(B)** with augmented images.

Figure 12 shows the performance metrics for DenseNet 121 implemented for with and without augmentation. The accuracy with augmented images is better than without augmented images. The AUC-ROC measure gives us a good idea of a model’s ability to differentiate between different classes. The more AUC a model has, the better it is judged to be. For every conceivable cut-off for a test or combination of tests, AUC-ROC curves are widely used to illustrate the relationship and trade-off between sensitivity and specificity. The accuracy for with and without implementing soft attention Map visualization is 95% and 99.62% is achieved.

The ROC curve’s area under the curve provides an indication of the test’s value in answering the underlying issue. At different threshold values, AUC—ROC curves may also be used as a performance evaluation. Using the AUC-ROC to assess the performance of a classification model is vital. When a model’s accuracy is improved *via* the use of this test, its value and correctness are both increased. In classification issues, the true positive rate and the predictive value of a predictive model may be summarized using this technique, which helps us understand the trade-off between the two. Table 4 compares the augmented and non-augmented on the scales of accuracy, sensitivity, specificity, precision and accuracy.

**TABLE 4:** Overall accuracy of DenseNet 121 for PD and control subject.

	Without augmented images					With augmented images				
Class	Accuracy	Sensitivity	Specificity	Precision	F1-Score	Accuracy	Sensitivity	Specificity	Precision	F1-Score
PD	94%	92%	91%	88%	88%	99%	99%	99%	99%	99%
CN	91%	89%	88%	86%	87%	99%	99%	98%	99%	99%
Overall Score	92.5%	90.5%	89.5%	87%	87.5%	99%	99%	98.5%	99%	99%

**FIGURE 13:**
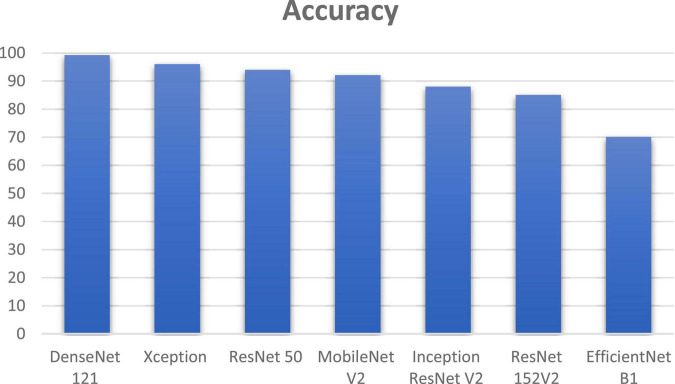
Comparison of DenseNet using soft attention with other deep learning algorithms.

DenseNet 121 gave an improvement of 3.2, 5.2, 7.2, 11.2, 14.2, and 29.2% in accuracy when compared to other deep learning algorithms such as Xception, ResNet 50, MobileNet V2, Inception ResNet V2, ResNet 152V2, and EfficientNet B1 as shown if Figure 13. DenseNet 121 solves the vanishing-gradient issue and encouraging feature reuse. DenseNet also decrease the number of parameters which yields an increase in accuracy.

In Table 5 the comparison of various deep learning techniques based on the computational time is listed. DenseNet 121 has the computational time of 2.15 min with an accuracy of 99.2%.

**TABLE 5:** Comparison of various deep learning models.

Deep learning algorithms	Computational time
Xception	1.5 min
ResNet 50	2.25 min
MobileNet V2	1.7 min
Inception ResNet V2	1.75 min
ResNet 152V2	3.3 min
EfficientNet B1	3.2 min
DenseNet 121	2.15 min

Many researchers have expanded their horizons by employing numerous deep learning frameworks to detect PD from normal and other disease categories, which might be used in future analysis and examinations. Magesh et al. (Adams et al., 2018) used transfer learning (with VGG16 as the leading model architecture) for classifying PD from normal groups. Local Interpretable Model-Agnostic Explainer (LIME) was selected as an illustratable method to find the region of interest to analyze PD and normal group DaTscan images. LIME proved to be vital substitute for explainable-AI based diagnosis to be used instead of Grad-CAM and saliency mapping representation.

Chien et al. (2020) reflected the focus on putamen and caudate region (from SPECT images) and demonstrated the use of artificial neural network for detecting PD and parkinsonism caused by other disorders. Sensitivity and specificity of 81.8% and 88.6% were achieved, though these research classes could be further investigated for study.

Nazari et al. (2022) tested layer-wise relevance propagation (LRP) based CNN for classification of normal and reduced patients. The study achieved a sensitivity and specificity of 92.8% and 98.7% respectively. Relevance maps were plotted which could be further investigated for clarity.

Chen et al. (2021) put focus on striatum scanning and implemented a CNN model based on attenuation correction. Monte-carlo based simulation results were drawn for a clearer visual assessment based on voxel-wise, patch-wise and image-wise imaging methods. Although computationally expensive, this strategy showed promise as a substitute in clinical scenario.

Tufail et al. (2021) attempted at developing 3D-CNN to extract attributes of Alzheimer’s Disease (AD), PD and normal classes from both PET and SPECT images. This multi-classification based experiment helped in establishing the relationship between AD and PD patients. This study reflects that 3D CNN models at relatively cheaper cost in computational levels could be thus developed for voxel based understanding of 3D SPECT images with explainable-AI based techniques implemented.

Leung et al. (2021) developed an approach by evaluating based on mean absolute error (MAE) and mean absolute percentage error (MAPE) for outcome prediction. The study was based on a three staged ensemble method to reveal spatiotemporal attributes, to demonstrate the connection between imaging and non-imaging information, for predictions based on motor outcomes.

The limitations of this study are, the selected features have been specifically tailored to the diagnosis of Parkinson’s disease. In people with Parkinson’s disease (PD), the illness advances in a predictable manner: First, the putamen on the side of the patient’s clinical symptoms begins to decline in DaT concentration, and subsequently the caudate. Striatum on DaTSCAN loses its comma-shape and becomes dot-shaped or vanishes completely when this occurs. It is possible to “force” a pre-defined region into the form of an exclamation point, resulting in a semi-quantitative metric that is still high but solely represents caudate binding, and therefore does not account for putamen dysfunction. When training, the ReLU may become unstable, resulting in the death of certain gradients. This is a serious drawback. So some neurons die and the weight updates don’t activate in subsequent data points, preventing learning since dead neurons offer zero activation.

## Conclusion

This work demonstrates that significant clinical examination performance may well be attained utilizing deep learning for SPECT scan interpretation and analyses. In order to accurately diagnose PD, it may be necessary to use DaTscan imaging to evaluate pathophysiological changes. Although our method allows to describe and visualize normal and PD cohorts relatively explicitly, of DaTscan SPECT images, using soft attention maps, it cannot be used for clinically analyzing the motor outcomes from SPECT images. Instead of using Gradient-weighted Class Activation Mapping (Grad-CAM), soft attention mapping is used which is cost-effective.

This is possible even with a modest number of participants by exploiting the strength of huge pre-trained neural networks through the transfer learning process along with manual addition of soft-attention block, as was done with DenseNet architecture in this study. The necessity for an end-to-end 3D CNN architecture should also be noted for future study. There were five CNN models employed in comparison with our CNN model: DenseNet 121, Xception 50, Resnet 50, Mobilenet V2, Inception ResNet V2, and EfficientNet B1. An AUC of 99% and an accuracy of 99.2 % are achieved in this system, compared to previously suggested methods. Further DenseNet-121 with the soft attention block retains features with low level of complexity.

This was a semi-automatic diagnostic process, not an entirely automated diagnosis monitoring system. Utilizing the complete scan volume rather than just a single slice may prove to be a rewarding topic of future study. If the transfer learning process is to be employed, this would need the usage of a properly pre-trained 3-D convolutional neural network. Though the simulation findings and study are intriguing, they can be corroborated with much bigger datasets. It is imperative to deal with the requirement for an end-to-end 3-D CNN model that can retrieve relevant features from the 3-D SPECT image data itself for improved clearer outcomes in future. This mandates that we continue to make progress on our research in deep learning and explainable-AI methods on Parkinsonism and related disorders.

## Data Availability Statement

The original contributions presented in this study are included in the article/supplementary material, further inquiries can be directed to the corresponding author/s.

## Ethics Statement

Ethical review and approval was not required for the study on human participants in accordance with the local legislation and institutional requirements. Written informed consent for participation was not required for this study in accordance with the national legislation and the institutional requirements.

## Author Contributions

HK was responsible for ideation, technique and design, data gathering and visualization, formal analysis, and reviewing and editing. MT conceptualized the project, developed the technique, designed the study, gathered and analyzed data, and then wrote the first draft. KL gathered and analyzed data as well as to write, evaluate, and edit the final product. SD and XW contributed to the formative stages of analysis and revision. All authors wrote the manuscript and gave their blessing to the final version that was submitted.

## Conflict of Interest

The authors declare that the research was conducted in the absence of any commercial or financial relationships that could be construed as a potential conflict of interest.

## Publisher’s Note

All claims expressed in this article are solely those of the authors and do not necessarily represent those of their affiliated organizations, or those of the publisher, the editors and the reviewers. Any product that may be evaluated in this article, or claim that may be made by its manufacturer, is not guaranteed or endorsed by the publisher.
